# The transcriptional regulator NtrC controls glucose-6-phosphate dehydrogenase expression and polyhydroxybutyrate synthesis through NADPH availability in *Herbaspirillum seropedicae*

**DOI:** 10.1038/s41598-017-12649-0

**Published:** 2017-10-19

**Authors:** Euclides Nenga Manuel Sacomboio, Edson Yu Sin Kim, Henrique Leonardo Ruchaud Correa, Paloma Bonato, Fabio de Oliveira Pedrosa, Emanuel Maltempi de Souza, Leda Satie Chubatsu, Marcelo Müller-Santos

**Affiliations:** Department of Biochemistry and Molecular Biology, Laboratory of Nitrogen Fixation, Federal University of Paraná (UFPR), Curitiba, Brazil

**Keywords:** Applied microbiology, Biopolymers

## Abstract

The NTR system is the major regulator of nitrogen metabolism in Bacteria. Despite its broad and well-known role in the assimilation, biosynthesis and recycling of nitrogenous molecules, little is known about its role in carbon metabolism. In this work, we present a new facet of the NTR system in the control of NADPH concentration and the biosynthesis of molecules dependent on reduced coenzyme in *Herbaspirillum seropedicae* SmR1. We demonstrated that a *ntrC* mutant strain accumulated high levels of polyhydroxybutyrate (PHB), reaching levels up to 2-fold higher than the parental strain. In the absence of NtrC, the activity of glucose-6-phosphate dehydrogenase (encoded by *zwf*) increased by 2.8-fold, consequently leading to a 2.1-fold increase in the NADPH/NADP^+^ ratio. A GFP fusion showed that expression of *zwf* is likewise controlled by NtrC. The increase in NADPH availability stimulated the production of polyhydroxybutyrate regardless the C/N ratio in the medium. The mutant *ntrC* was more resistant to H_2_O_2_ exposure and controlled the propagation of ROS when facing the oxidative condition, a phenotype associated with the increase in PHB content.

## Introduction

Poly-3-hydroxybutyrate (PHB) is an aliphatic polyester member of the polyhydroxyalkanoates (PHA) family synthesised by some bacteria as carbon and reducing equivalents storage^1,2^. Usually, bacteria produce PHB under conditions of carbon excess and low levels nitrogen, phosphate and oxygen^3^. At least three enzymes are involved in its synthesis: 3-ketothiolase, acetoacetyl-CoA reductase and PHA synthase encoded by *phaA*, *phaB* and *phaC* respectively^4^. These enzymes catalyse the condensation of acetyl-CoA forming acetoacetyl-CoA, then reduction of acetoacetyl-CoA to 3-hydroxybutyryl-CoA (3HB-CoA) and finally polymerisation of 3HB-CoA to yield PHB^5^.

PHB is a thermoplastic biodegradable polymer with physicochemical properties comparable to recalcitrant oil-based plastics such as polypropylene and polystyrene^6^. Although PHB is a sustainable alternative to such plastics, its production cost is still considerably higher, creating a necessity for engineering of PHB overproducer strains and process optimisation. So far, several studies of metabolic engineering for improving microbial PHB production have been reported^7–11^. The majority of these studies focused on carbon metabolism pathways engineering and the improvement of NAD(P)H availability. Although the carbon to nitrogen (C/N) ratio is a major factor controlling PHB accumulation in several bacteria^12^, little attention has been given to re-engineering the nitrogen metabolism. In bacterial cultivation for the production of PHB, the addition of high ammonium concentration to the medium improves the cell growth but reduces the production of PHB^13^. The negative effect is due to that with a high concentration of ammonium the bacterium diverts much of the carbon skeleton to produce amino acids and other nitrogenous molecules. Therefore, a better understanding of the carbon and nitrogen metabolism interrelationships can contribute to engineering better PHB producers.

**Figure 1:**
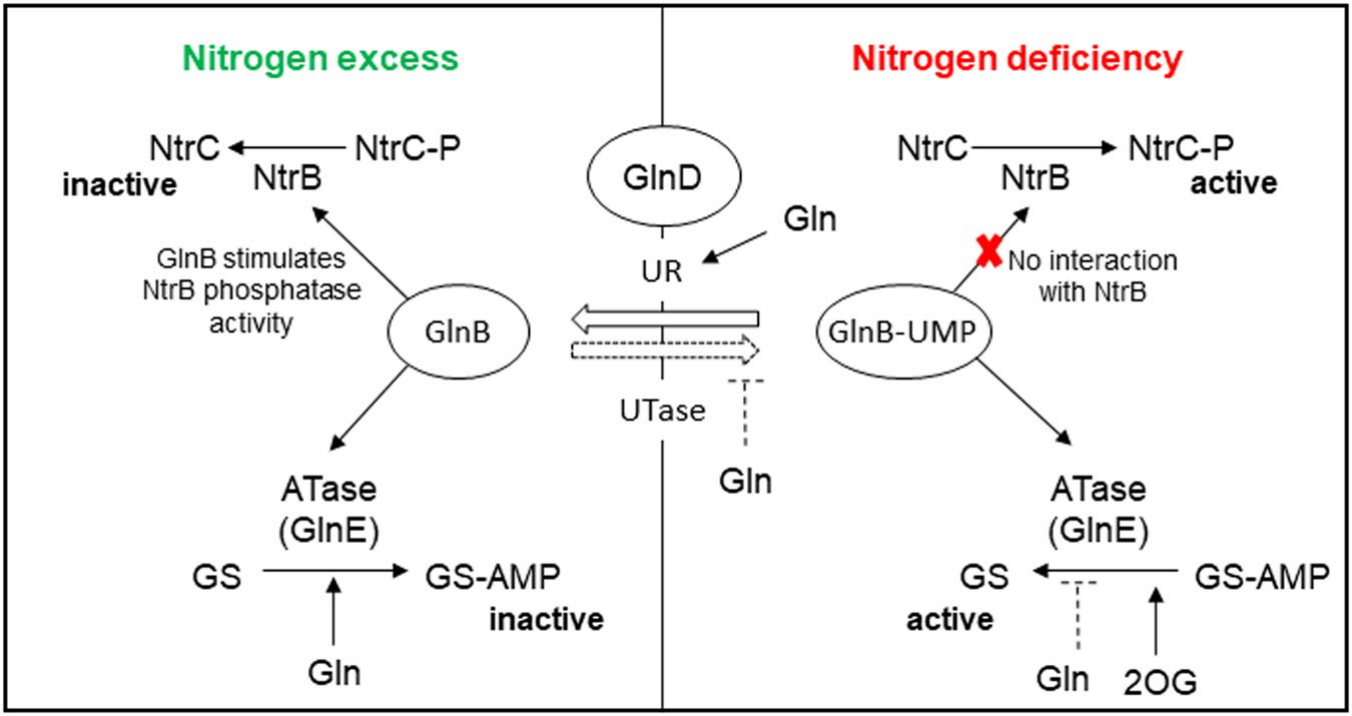
The NTR system and its regulation. In nitrogen excess, high glutamine concentration activates the deuridylylation of GlnB by GlnD. The unmodified form of GlnB stimulates the adenylyl transfer activity of GlnE, resulting in glutamine synthetase adenylation (GS-AMP) and therefore its inactivation. GlnB also interacts with the NtrB stimulating its phosphatase activity, resulting the dephosphorylation of the transcriptionally active form of NtrC. On the other hand, when the bacterium faces a nitrogen-limiting condition, the low glutamine concentration leads to the uridylylation of GlnB (GlnB-UMP) by GlnD. GlnB-UMP stimulates the deadenylylation of GS-AMP, turning GS active. The 2-oxoglutarate (2-OG) is an effector of GlnB, and its concentration is high under nitrogen-limiting conditions.

*Herbaspirillum seropedicae* SmR1 is a diazotrophic β-Proteobacterium that associates beneficially with economically relevant species of Poaceae^14^ and produces PHB as means of carbon and energy storage^15–17^. Thirteen genes probably involved in PHB metabolism were identified in *H. seropedicae* SmR1 by genome analysis^18^, including four *phaC*, two *phaZ* and two *phaP* genes, encoding PHA synthases, PHA depolymerases and phasins, respectively. The PHB synthesis in *H. seropedicae* SmR1 seems to be entirely supported by the PHA synthase encoded by *phaC1* since its deletion abolishes PHB accumulation^19^. The PHA depolymerases are the enzymes that mobilise the PHB granules releasing 3-hydroxybutyrate (3HB) or oligos of 3HB. The phasins are the proteins more abundant on the surface of PHB. They are important to avoid the coalescence of the granules and also to control their number and size. Recently, the role of the two phasins (PhaP) was described in *H. seropedicae* SmR1, showing that PhaP1 is the main phasin and highly expressed in conditions of PHB production, on the other hand, PhaP2 acts as backup phasin when *phaP1* is not expressed^17^. The nitrogen metabolism and its regulation have been extensively studied in *H. seropedicae* SmR1^20^, turning *H. seropedicae* a model to investigate the impact of C/N ratio on PHB production. Specifically, several components of the NTR system, the master nitrogen regulatory system, were studied in *H. seropedicae* SmR1. The NTR system is composed of a cascade of regulatory proteins controlling the nitrogen assimilation in Bacteria^21^ (Fig. 1). Five proteins are involved: GlnD, a uridylyl transferase/uridylyl-removing enzyme; the signal-transducing proteins GlnB (PII) and GlnK; NtrB, a histidine protein kinase; and the response regulator, NtrC^21,22^. Under limiting nitrogen conditions, GlnD uridylylates PII which as PII-UMP enables NtrB to phosphorylate NtrC. Phosphorylated NtrC positively activates several nitrogen assimilation operons dependent of the sigma 54 (RpoN) factor, including *glnAntrBntrC*^23^. Under high nitrogen conditions, GlnD deuridylylates PII-UMP, activating in turn, the dephosphorylation of NtrC by NtrB and hence rendering it inactive. The influence of the NTR system on carbon metabolism, specifically on PHB metabolism, was already studied in *Azospirillum brasilense* Sp7^24,25^. The authors found that the *ntrC* mutant could produce PHB in both low- and high-C/N ratio media, while the wild-type had no significant PHB production in low-C/N ratio^24^. Also, mutants in both PII proteins genes (*glnB* and *glnZ*) or *glnD* had higher contents of PHB than the wild type under low-C/N ratio^25^. These findings indicate that PHB synthesis is coupled with the nitrogen levels via the components of the NTR system. To better understand how the NTR system affects PHB production in bacteria, we investigated the PHB synthesis in NTR mutants of *H. seropedicae* SmR1. The results obtained in this work reveal a new facet of how the NTR system can influence the carbon metabolism, especially in the synthesis and accumulation of PHB.

## Results

### The *ntrC* mutant of *H. seropedicae* SmR1 produces more PHB than the parental strain

**Figure 2:**
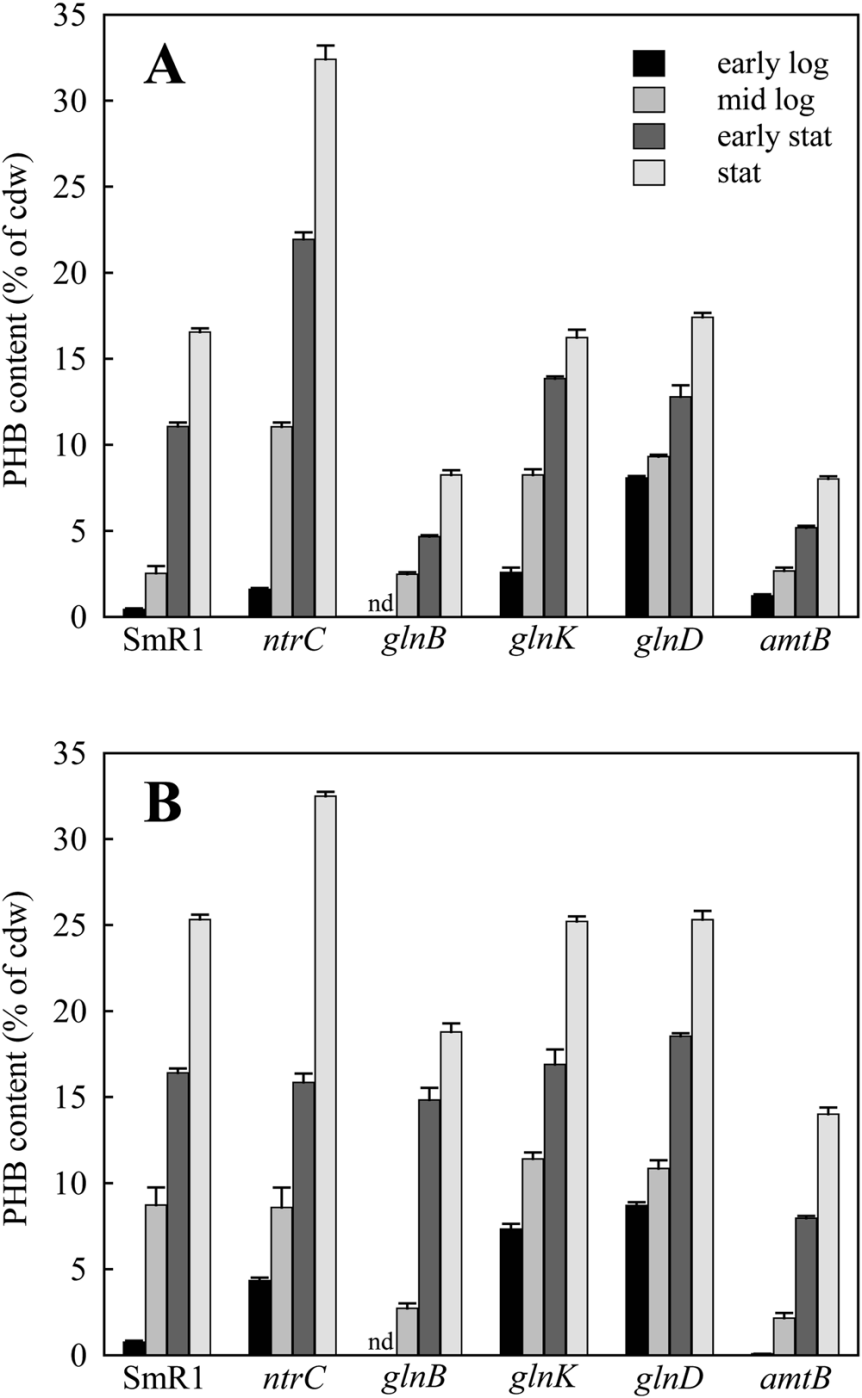
PHB accumulation profiles of *H. seropedicae* SmR1 (parental strain) and the NTR system mutants. Strains grew in NFbHP medium amended with 37 mM DL-malate and 20 mM (**A**) or 5 mM (**B**) of ammonium chloride at 30 °C (orbital agitation at 120 rpm). The PHB contents were determined in three independent samples. nd – non-detected.

The C/N ratio is one of the key factors controlling PHB accumulation in bacteria^26,27^. Accordingly, we anticipated that mutant strains of the NTR system would be a useful tool to investigate its involvement in PHB synthesis. We measured the content of PHB in *glnB*, *glnK, glnD, amtB* and *ntrC* knock-out mutants as well as in the parental strain *H. seropedicae* SmR1. The strains were grown in low C/N ratio (37 mM DL-malate and 20 mM NH_4_Cl) and high C/N ratio (37 mM DL-malate and 5 mM NH_4_Cl). The cultures were sampled in four stages of growth: early-exponential phase (OD_600_ 0.4), mid-exponential phase (OD_600_ 0.8), early stationary phase (OD_600_ 1.2) and late stationary phase (OD_600_ 1.6). The PHB contents of the parental, *glnK* and *glnD* strains were very similar since their maximum PHB content was around 25%/cdw (cell dry weight) in high C/N (Fig. 2). The PHB was reduced to 16%/cdw when the strains grew in low C/N. Interestingly, the *glnB* and *amtB* mutants presented a significant reduction of PHB accumulation compared to the parental strain (Fig. 2). This effect was remarkable in low C/N where *glnB* and *amtB* mutants produced only half of the PHB level of the parental strain. In contrast, PHB production in the *ntrC* mutant was unregulated and presented the highest content of PHB amongst all strains analysed, reaching 32% of PHB regardless the initial C/N ratio (Fig. 2). This higher PHB production regardless the nitrogen level in the medium suggests that *ntrC* knock-out decouples the synthesis of PHB of the C/N ratio.

### The *ntrC* mutant also produces more PHB with monosaccharides as carbon source

**Figure 3:**
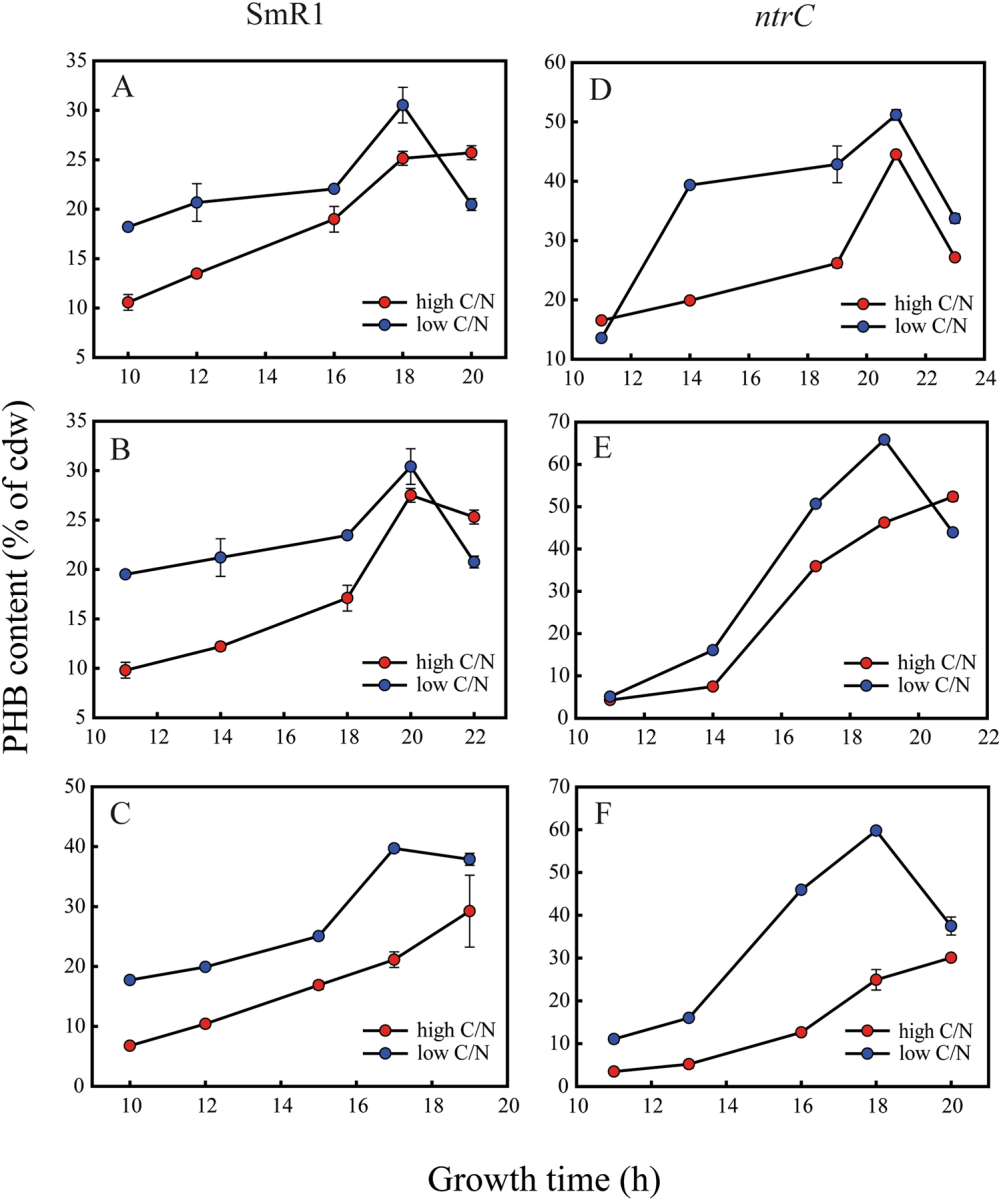
PHB accumulation profiles of *H. seropedicae* SmR1 and *ntrC* mutants in D-glucose, D-fructose and D-xylose as sole carbon sources. Strains grew in NFbHP medium amended with 25 mM D-glucose (**A** and **D**), 25 mM D-fructose (**B** and **E**) or 30 mM D-xylose (**C** and **F**). Low C/N ratio media had 20 mM NH_4_Cl (blue circles), while high C/N ratio media had 5 mM NH_4_Cl (red circles). PHB contents were determined in three independent samples. Strains were cultivated at 30 °C at 120 rpm (orbital shaking).

As malate is not a conventional substrate for biotechnological applications due to its high price compared to other sources, we measured PHB accumulation in the *ntrC* mutant on D-glucose and D-xylose, which are monosaccharides highly abundant in cheap feedstocks and agro-industrial residues. The C/N ratios were the same. When grown in the presence of D-glucose, the maximum PHB production of the *ntrC* mutant was 1.8- and 1.7-fold higher than the parental production, in low and high C/N ratio, respectively (Fig. 3A and D). On D-fructose, the maximum PHB production of the *ntrC* mutant was 2.0- and 2.15-fold higher than the parental production, in low and high C/N ratio respectively (Fig. 3B and E). Interestingly, when D-xylose was used as the carbon source, in low C/N ratio there was no difference of PHB between both strains, but in high C/N ratio, the *ntrC* mutant produced 1.5-fold more PHB (Fig. 3C and F). Table 1 shows the maximum content of PHB and the productivities obtained for both strains in the different conditions assayed. The profile of sugar consumption and the data of PHB concentration (g/L) and yield of g PHB/g of substrate for the SmR1 and *ntrC* strains are shown in the supplemental information (Figure S1 and Tables S1 and S2).

**Table 1:** Maximal PHB productivities of the parental strain SmR1 and the *ntrC* mutant of *H. seropedicae* SmR1.

Strain	Carbon Source	Parameter	Nitrogen concentration (NH_4_Cl)	
			5 mM	20 mM
SmR1	Glucose	CDW (mg/Lh)	30.6	38.3
		PHB (mg/Lh)	12.4	11.5
		% PHB	40.4 ± 1.8	30.1 ± 0.7
	Fructose	CDW (mg/Lh)	28.9	36.1
		PHB (mg/Lh)	8.8	9.0
		% PHB	30.5 ± 1.8	25.2 ± 0.7
	Xylose	CDW (mg/Lh)	37.0	36.9
		PHB (mg/Lh)	14.7	10.8
		% PHB	39.7 ± 0.6	29.2 ± 6
*ntrC*	Glucose	CDW (mg/Lh)	53.3	47.3
		PHB (mg/Lh)	27.3	21.1
		% PHB	51.2 ± 0.9	44.5 ± 0.2
	Fructose	CDW (mg/Lh)	47.4	55.7
		PHB (mg/Lh)	31.2	29.2
		% PHB	65.8 ± 0.7	52.3 ± 1
	Xylose	CDW (mg/Lh)	29.5	38
		PHB mg/Lh	17.6	11.5
		% PHB	59.8 ± 0.2	30.1 ± 0.5

### Complementation of the *ntrC* mutant restores PHB production to the parental level

**Figure 4:**
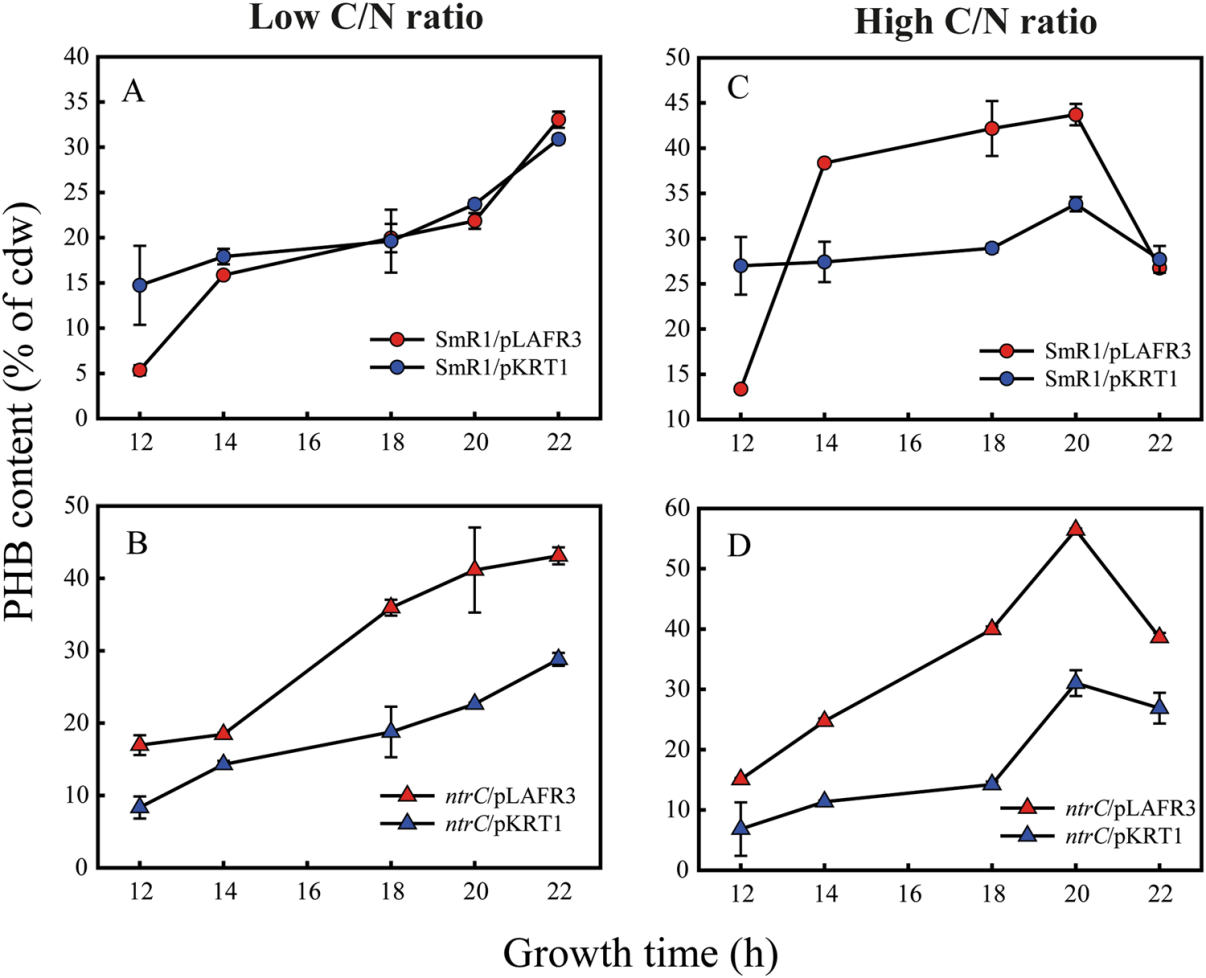
PHB content of *ntrC* mutant strain carrying an additional copy of the operon *glnAntrBntrC* of *H. seropedicae* SmR1. Strains harbouring the plasmids pLAFR3 (empty vector) or pKRT1 (pLAFR3 with the *glnAntrBntrC* as an insert) grew in NFbHP medium amended with 25 mM D-glucose. Graphs A and C represent the data for SmR1, while B and D for the *ntrC* mutant at low and high C/N ratio, respectively. Red symbols are non-complemented strains, while blue symbols correspond to the complemented ones. Data represent the average ± standard deviation of three independent samples.

The *ntrC* gene is clustered in an operon downstream from *glnA* and *ntrB*^18,28^. The *ntrC* mutant was complemented through pKRT1 conjugation. The pKRT1 is a pLAFR3-derivative containing a *glnAntrBC* operon copy from *H. seropedicae* SmR1^23,29^. PHB accumulated in strains harbouring pKRT1 was reduced when grown in malate or glucose, regardless the C/N ratio applied (Fig. 4). Therefore, the complementation of the *ntrC* mutant demonstrates that the NtrC is directly involved in the higher PHB production observed.

### The derepression of the *zwf* (glucose-6-phosphate dehydrogenase) gene results in high NADPH in the *ntrC* mutant

**Figure 5:**
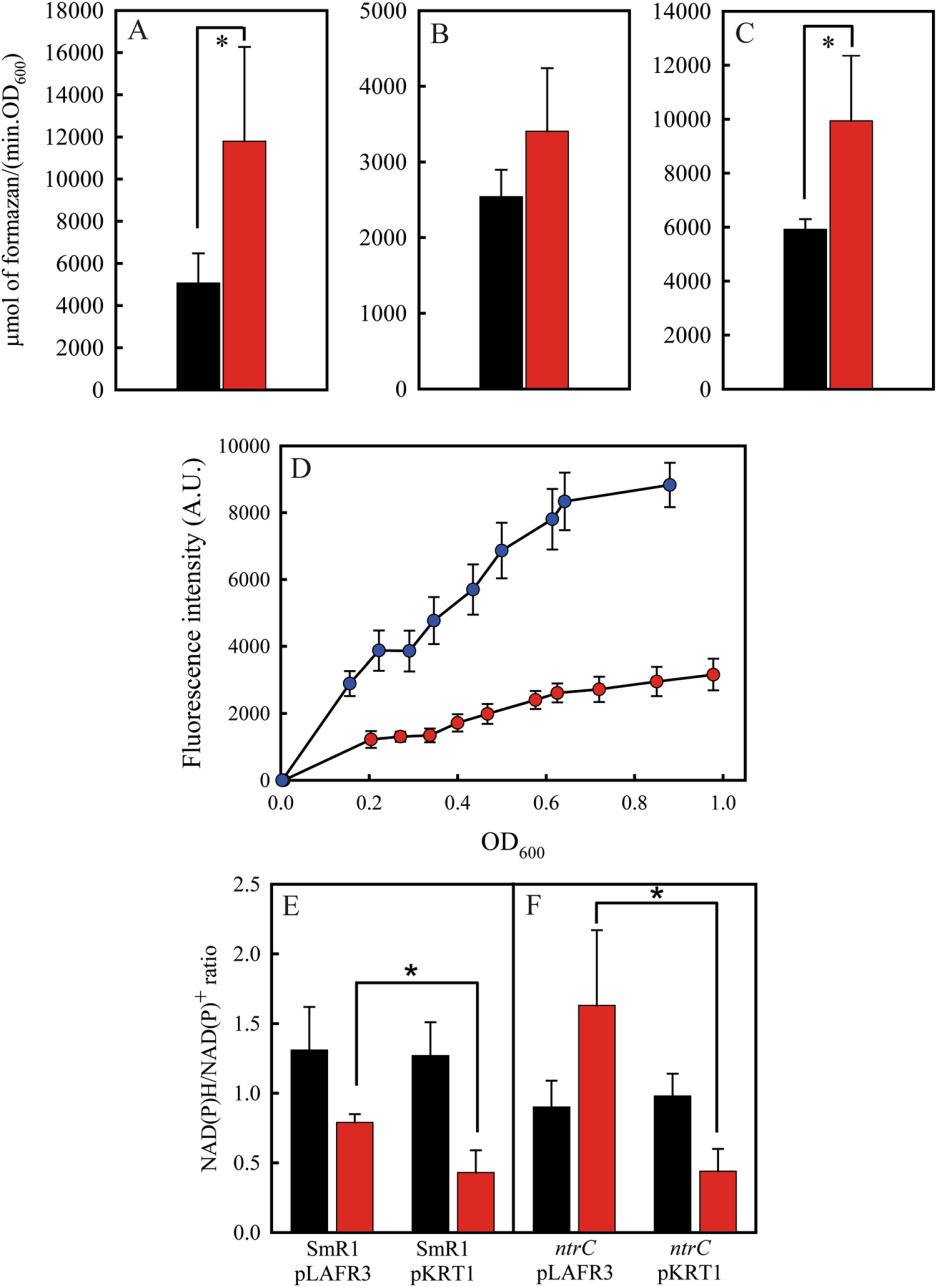
The *ntrC* mutant presents increased G6PDH activity and NADPH/NADP^+^ ratio. The G6PDH (**A**), GDH (**B**) and ME (**C**) activities were measured in crude extracts of the SmR1 (black bars) and *ntrC* (red bars) strains cultivated at 30 °C in NFbHP with 25 mM of glucose and 20 mM of ammonium chloride. (**C**) The transcriptional activity of the P*pgi*-*gfp* fusion was measured during SmR1 (red circles) and *ntrC* mutant (blue circles) cultivation at 30 °C in NFbHP with 25 mM of glucose and 20 mM of ammonium chloride. The NADH/NAD^+^ (black bars) and NADPH/NADP^+^ (red bars) ratios were measured for strains SmR1 (**E**), *ntrC* (**F**) and both strains complemented with pKRT1. Where appropriate, statistical significance is shown (*−p-value ≤ 0.05, independent two-sample t-test).

Previous works have shown that NtrC is involved in the regulation of the expression of glucose-6-phosphate dehydrogenase (G6PDH) and glutamate dehydrogenase (GDH)^30,31^. Specifically, the G6PDH activity is widely implicated in the generation and maintenance of the NADPH/NADP^+^ ratio^32,33^. The activity of the NADP^+^-dependent malic enzyme (ME) is also correlated with NADPH/NADP^+^ balance^34^. This observation led us to determine the activity of these enzymes in the parental strain SmR1 and *ntrC* mutant. The G6PDH and NADP^+^-dependent ME activities were 2.3- and 1.6-fold higher in the *ntrC* mutant when grown in NFb-glucose with 20 mM NH_4_Cl (Fig. 5A and C). GDH activity did not differ statistically between both strains (Fig. 5B). The specific activities of G6PDH, ME and GDH enzymes for the SmR1 and *ntrC* strains are shown in the Supplemental Figure S2. The repression of *zwf* transcription by NtrC has been previously reported for the bacterium *Pseudomonas putida*^3,30^. The *zwf* of *H. seropedicae* is located downstream from *pgi* (Hsero1099, encoding a phosphoglucoisomerase) and upstream from a gene encoding a transcriptional regulator of the HexR family (Hsero1097) and *talB* (Hsero1096, encoding a transaldolase). Since the 76 bp region between *pgi* and *zwf* seems not to contain a promoter region, the upstream region of *pgi* was cloned with GFP in the pEKGFP01 to determine the expression profile of the operon carrying *zwf* in *H. seropedicae* SmR1 and *ntrC* mutant. The activity of the P*pgi*-*gfp* fusion in the *ntrC* mutant was higher than that of the parental strain during all growth phases, achieving a maximum difference of 2.8-fold (Fig. 5D). The transcription of *zwf* and other genes involved in the Entner-Doudoroff pathway and in the PHB metabolism were compared between the SmR1 and *ntrC* strains through RNA-seq analysis. The data corroborate the higher expression of *zwf* in the *ntrC* mutant (Table S3). Also, the transcription of genes involved in the PHB metabolism was lower in the *ntrC* mutant (Table S4), indicating that the higher PHB production measured is a consequence of a metabolic factor. This prompted us to measure the NAD(P)H/NAD(P)^+^ ratio in both strains. The NADH/NAD^+^ ratio had no statistically significant difference between the parental strain SmR1 complemented or not with an additional copy of the operon *glnAntrBntrC* cloned into pLAFR3 (pKRT1) (Fig. 5E). The same was observed for the NADH/NAD^+^ ratio in the *ntrC* mutant (Fig. 5F). However, the NADPH/NADP^+^ ratio was statistically higher for both strains when they were not complemented with pKRT1. For the *ntrC* mutant, the NADPH/NADP^+^ ratio was 2.1-fold greater than in the parental strain (Fig. 5E and F, comparing the red bars). Taken together these results indicate that the knock-out in *ntrC* increases the expression of G6PDH and the regeneration of NADP^+^ in NADPH.

### The *ntrC* mutant of *H. seropedicae* SmR1 is more resistant to oxidative stress caused by hydrogen peroxide

**Figure 6:**
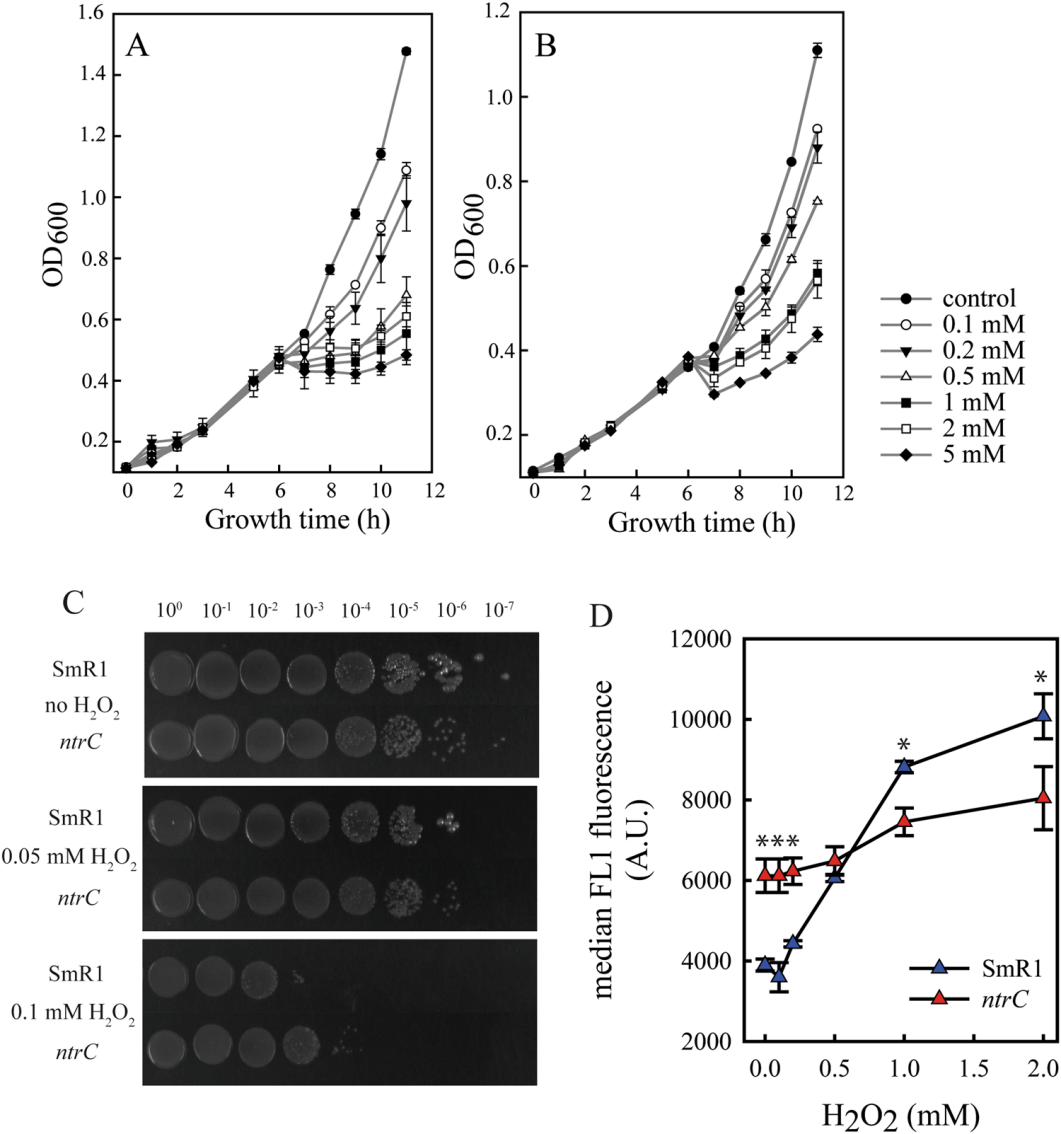
The effect of H_2_O_2_ on growth and ROS propagation in the parental strain and the *ntrC* mutant of *H. seropedicae* SmR1. Growth kinetics of parental strain SmR1 (**A**) and *ntrC* mutant (**B**) in NFbHP-malate medium in the presence of increasing hydrogen peroxide concentrations. The growth kinetics were determined in three independent samples for each strain. At the time of 6 h hydrogen peroxide was added to the cultures. (**C**) SmR1 and *ntrC* mutant grew to stationary phase and serial dilutions were plated onto NFbHP-malate medium containing H_2_O_2_ at the indicated concentrations. After two days of incubation at 30 °C, the SmR1 strain presented higher hypersensitivity to H_2_O_2_ than the *ntrC* mutant. (**D**) Median fluorescence of H_2_-DCFDA treated cultures during H_2_O_2_ stress in SmR1 and *ntrC* strains. The median fluorescence was determined in three independent samples for each strain. Where appropriate, statistical significance is shown (*−p-value ≤ 0.05, independent two-sample t-test).

A high NADPH/NADP^+^ ratio stimulates PHB production^35,36^. High [NADPH] increases the flux towards PhaB (NADPH-dependent acetoacetyl-CoA reductase), yielding PHB not only as a carbon stock but also a redox sink^2,36^. Furthermore, NADPH is crucial to anti-oxidative defences in most organisms, ensuring a reductive cellular environment to mitigate the deleterious effects of oxidative species as hydrogen peroxide, hydroxyl radical and superoxide^37^. Therefore we determine the resistance to oxidative stress of both strains. Cellular growth was assessed in media amended with increasing concentrations of hydrogen peroxide (H_2_O_2_). The addition of the oxidant at 6 h of cultivation impaired the growth of the parental strain in concentrations above 0.2 mM, while the *ntrC* mutant resisted the oxidative shock, recovering growth after one hour even at 5 mM of H_2_O_2_, as observed in Fig. 6B. The serial dilution onto agar plates containing H_2_O_2_ showed that the *ntrC* mutant was able to grow in dilution of 10^4^-fold while the parental SmR1 growth only until 10^2^-fold dilution at 0.1 mM H_2_O_2_ (Fig. 6C, lower panel). The ROS measurement applying the fluorescent probes H_2_-DCDFDA showed that the *ntrC* mutant was able to control ROS propagation, while the parental strain could not maintain the ROS at low levels (Fig. 6D). The fluorescence value of the oxidised H_2_-DCDFDA increased 2.5-fold from 0 to 2 mM of H_2_O_2_ in the parental SmR1, while in the *ntrC* mutant the oxidation of the probe increased 1.3-fold applying the same treatment with H_2_O_2_. Taken these results together, we conclude that the *ntrC* mutant has a more efficient defence against the oxidative stress, probably due to the NADPH accumulation which can be applied to mitigate the deleterious effects of the oxidative insult. The high NADPH generation also stimulated the PHB synthesis observed in the mutant.

## Discussion

The nitrogen level in the growth medium is a key factor interfering with PHB production in bacteria^38–40^. Therefore, the interruption of nitrogen regulatory systems can be a useful strategy to reduce the C/N ratio effect on PHB production and ultimately to improve PHB production. Since the NTR system is the master regulator of the nitrogen metabolism in several bacterial species^41^, wherein we investigate the PHB accumulation using as a model the bacterium *H. seropedicae* SmR1 and a set of mutants defective in the expression of regulatory proteins of the NTR system. Among all strains evaluated only the *ntrC* mutant produced higher PHB contents than the parental strain. The *ntrC* mutant produced around the double of PHB than the parental strain (in % PHB/mg of cdw), in all tested conditions. Similar results were also reported to *ntrB* and *ntrC* mutants of *A. brasilense* Sp7^24^, suggesting that possibly the effect of NtrC on PHB synthesis is widespread among prokaryotes. Based on these findings, we anticipated some possible explanations for our results.

The synthesis of PHB is largely dependent on high levels of acetyl-CoA and NADPH. Normally, both metabolites are in high intracellular concentrations when the bacterium faces a condition of carbon excess and limitation in another nutrient, such as nitrogen^1^. This condition is permissive for PHB synthesis due to elevating acetyl-CoA level generated by carbon overflow and high NADPH/NADP^+^ ratio as consequence of nitrogen limitation^7,9,35^.

Considering that *H. seropedicae ntrC* mutant produced more PHB than the parental strain even under unfavourable conditions (low C/N ratio) suggests that NtrC somehow is modulating acetyl-CoA and/or NADPH concentrations. A role of NtrC on directly activating transcription of genes involved in PHB synthesis is unlikely since PHB production is higher in the *ntrC* mutant even under an excess of ammonium, a condition where NtrC is mainly dephosphorylated and therefore inactive^22^ (Fig. 2A and B). A transcriptomic analysis of the ∆*ntrC* mutant of the PHA-producing bacterium *Pseudomonas putida* KT2442 brought important elements to understand the link between NtrC and PHA metabolism^3^. In the ∆*ntrC* strain, the transcription of *zwf-1* (encoding glucose-6-phosphate dehydrogenase – locus tag PP1022) and *gap-1* (glyceraldehyde-3-phosphate dehydrogenase - PP1009) was upregulated 5.7- and 2.6-fold, indicating that NtrC represses their expression. Hervás *et al*. suggested that repression of *zwf* shows that NtrC controls hexose catabolism in bacteria, likely to prevent a carbon overflow under nitrogen-limiting conditions for growth^3^. In another work, the *ntrC* deletion in *P. putida* KT2440 also rendered cells more resistant to oxidative stress^30^. Furthermore, *zwf-1* (PP1022) was also up-regulated in the ∆*ntrC* mutant of *P. putida* KT2440, as determined by transcriptomic analysis. *H. seropedicae* SmR1 and *P. putida* have the same incomplete glycolytic pathway since it lacks 6-phosphofructokinase (PFK-1). As a consequence, the Entner-Doudoroff (ED) pathway converts glucose and fructose into pyruvate and glyceraldehyde-3-phosphate. Particularly, in mutants defective in *ntrC* expression, it is expected a higher metabolic flux through glucose-6-phosphate dehydrogenase, generating more acetyl-CoA and NADPH, which in turn increases PHB accumulation.

These findings suggest that the up-expression of *zwf* could be a major factor leading *ntrC* defective mutants to accumulate more PHB under permissive conditions. The observation that the *H. seropedicae ntrC* mutant produced more PHB even in unfavourable conditions (low C/N ratio) suggests a higher pool of reducing power as compared to the parental strain. Therefore is likely that the bacterium switches the synthesis of PHB as an electron sink to avoid the deleterious effect of a high reductive environment.

In fact, we have already reported that mutant strains defective in PHB synthesis or accumulation presented a severe growth penalty on glucose^17^. Probably, as a consequence of the redox imbalance caused by the inability of those mutants to divert electrons towards PHB synthesis.

NtrC is widely recognised as a transcriptional regulator restricted to regulate genes involved in nitrogen metabolism. Indeed, a significant portion of genes corresponding to 2% of the *E. coli* genome was determined to be under control of the NtrC transcriptional activity, most of them involved in amino acids and ammonium transport, nitrogen assimilation and nitrogen recycling^42^. Our results demonstrate that NtrC interferes with PHB synthesis, bring to the discussion if NtrC could be one of the connection points between nitrogen and carbon metabolism. Alternatively, the NtrC control of the NADPH recycling may help cells dealing with a stressful condition such as nitrogen limitation. In fact, the glutamate synthase (GOGAT) and the glutamate dehydrogenase (GDH), which are important reactions for ammonium assimilation require NADPH as an electron donor to catalyse the amination of 2-oxoglutarate^43^. In *E. coli*, NADPH concentration was homeostatic after ammonium upshift^44^. Since NADPH consumption increases when ammonium assimilation is higher, it is likely that other pathways are generating the NADPH demanding^44^. Whether or not the phosphorylation state of NtrC interferes in NADPH production through *zwf* expression is uncertain. The repression of *gdhA* encoding the glutamate dehydrogenase in *P. putida* KT2442 by NtrC was shown to be independent of the phosphorylation state, since both wild-type and the mutant NtrC^D55E,S161F^ (mimicking the phosphorylated protein) can bind to the *gdhA* promoter and repress transcription^31^. The effect of phosphorylation could be further studied employing a *ntrB* mutant of *H. seropedicae* and complementation of the *ntrC* mutant with NtrC variants unable to be phosphorylated. Such experiments will determine if the reduction in PHB production observed in the *glnB* and *amtB* mutants derived from the phosphorylation state of NtrC.

It would be interesting to investigate further the role of NtrC on PHB synthesis in other PHB-producing models, such as *Ralstonia eutropha*, *Azotobacter vinelandii* and *P. putida*. It would point if the involvement of NtrC on PHB synthesis is conserved among other classes of bacteria, serving as a strategy for metabolic engineering aiming to improve PHB production.

## Methods

### Bacterial Strains, Plasmids, and Growth Conditions

Strains and plasmids used are listed in Table 2. *Escherichia coli* strain Top10 (Thermo Fisher Scientific Inc., Waltham, MA, USA) and *E. coli* S17-1^45^ were used for cloning and conjugational procedures, respectively. *E. coli* strains grew in LB medium at 37 °C and shaken at 160 rpm. *H. seropedicae* parental strain SmR1^46^ and mutant strains were cultured in NFbHP media with 37 mM DL-malate and the indicated concentration of NH_4_Cl at 30 °C and shaken at 120 rpm^49^. D-glucose at 25 mM, D-fructose at 25 mM and D-xylose at 30 mM were applied as alternative carbon sources for PHB production measurement as a replacement for malate. Two regimes of carbon-to-nitrogen (C/N) ratio were used at the start of cultivation: high C/N ratio with 5 mM of NH_4_Cl and low C/N with 20 mM of NH_4_Cl.

**Table 2:** Bacterial strains and plasmids used in this work.

Strain or plasmid	Relevant characteristics	Reference/source
*E. coli*		
Top10	Cloning host	Thermo Fischer Scientific
S17-1	Conjugational transfer of plasmids	45
*H. seropedicae*		
SmR1	Parental strain, Nif^+^, Sm^R^	46
*ntrC* (DCP286A)	SmR1 containing *ntrC*::Tn5-B20	23
*glnB*	SmR1 containing *glnB*::Tc^R^	47
*glnK*	SmR1 with a chromosomal deletion of the *glnK*	47
*glnD*	SmR1 containing *glnD*::Tc^R^	Unpublished result
*amtB*	SmR1 containing *amtB*::*lacZ*-Km^R^	47
Plasmids		
pBBR1MCS-3		48
pLAFR3	Broad-host-range cloning vector, IncP1, Tc^R^	29
pKRT1	23-kb fragment from *H. seropedicae* SmR1 harbouring the *glnA*, *ntrB* and *ntrC* genes	46
pEK07	pBBR1MCS-3 containing *pgi* upstream region (P*pgi*) from *H. seropedicae* SmR1 cloned between XhoI and SpeI restriction sites	This work
BBa_I13504	Plasmid pSB1C3-derivative containing the *gfp*mut3b gene (BBa_E0040) with the strong rbs (BBa_B0034) and the double terminator (BBa_B0015).	Registry of Standard Biological Parts (partsregistry.org)
pEKGFP01	pBBR1MCS-3 containing *pgi* upstream region (P*pgi*) from *H. seropedicae* SmR1 and *gfp*mut3b downstream.	This work

### Quantification of PHB

PHB was quantified by methanolysis, followed by GC-FID (gas chromatography coupled to a flame ionisation detector) analyses as described previously^50^ with 5 to 10 mg of lyophilised bacteria. Amounts of PHB in each sample were normalised to cell dry weight (cdw; the weight of the lyophilised bacterial pellet) and expressed as % of PHB cell dry weight^−1^.

### Construction of P*pgi-gfp* transcriptional fusion

The intergenic region of the *pgi* (locus-tag Hsero1099) was amplified using the primers Fw_Ppgi_Hs 5′TATCTCGAGTGTCGGGTTCCTGTTAGCGT 3′, containing a XhoI site (underlined) and Rev_Ppgi_Hs 5′TATACTAGTCATATGGGTCTGGTGTCGGTTGGCGG 3′, containing a SpeI site (underlined) as previously described^51^. The amplified product was cloned into the sites XhoI and SpeI of the pBBR1MCS-3^52^, generating the pEK07. The reporter gene *gfp* containing upstream the rbs site B0034 and downstream the double terminator B0015 was extracted from the plasmid BBa_I13504 (Registry of Standard Biological Parts, partsregistry.org) digested with the EcoRI and SpeI enzymes and cloned into pBlueScript II KS(+) digested with EcoRI and XbaI. Then, the *gfp* cassette was removed by digestion with the XbaI and SacI enzymes and cloned into the pEK07 digested with the same enzymes, generating the pEKGFP01. The pEKGFP01 was transformed in *E. coli* S17-1 and conjugated to *H. seropedicae* by bi-parental mating.

### Measurement of P*pgi-gfp* transcriptional activity

To measure the transcriptional activity of the *pgi* promoter, 2 μL of saturated cultures containing either the transcriptional fusion pEKGFP01 or an empty pBBR1MCS-3 vector were inoculated in a 96-well microplate containing 200 μL of NFbHP-glucose with 20 mM of NH_4_Cl as a nitrogen source. The microplate was incubated in an orbital shaker (Incubator Shaker Series I26, New Brunswick™) at 30 °C and 120 rpm. The fluorescence was measured using a Berthold™ TriStar LB 941 using 355 nm filter for excitation and a 535 nm for the emission wavelength. Arbitrary fluorescence units were normalised by OD readings at 600 nm using a Bio-Rad iMark™ Microplate Reader.

### Complementation of *ntrC* mutant

The pKRT1 cosmid was conjugated by bi-parental mating between *E. coli* S17-1 and *H. seropedicae* strains. The transconjugant colonies were selected in NFbHP-malate with 20 mM NH_4_Cl agar with 10 µg/mL of tetracycline. The complemented strains were cultivated in NFbHP-malate and NFbHP-glucose at high and low-C/N ratios.

### Determination of enzymatic activities

Glucose-6-phosphate dehydrogenase (G6PDH), glutamate dehydrogenase (GDH) and malic enzyme (ME) activity assays were performed by measuring the formazan production at 585 nm, as previously described^52^. Formazan is the insoluble product formed by MTT (3-(4,5-dimethyl-thiazolyl-2)-2,5-diphenyl-tetrazolium bromide) reduction. Cells were lysed by sonication on an ice bath. The lysates were centrifuged at 4,000 × g for 10 min at 4 °C. The supernatants were maintained on ice until assay setup. The reaction contained 300 μM NADP^+^, 300 μM of the substrate (D-glucose-6-phosphate, DL-malate or L-glutamate), 300 μM MTT and 30 μM PES (phenazine ethosulphate). The components were dilute up to 900 µL in 50 mM Tris-HCl buffer at pH 8 with 0.13% (m/v) gelatin. The gelatin was employed to prevent the formazan precipitation. The reactions were carried out in 1 mL-cuvettes, starting by addition of 100 μL of the supernatant. The reactions were monitored for 5 min at 585 nm in a Shimadzu™ spectrophotometer. The activity was expressed as µmol of formazan/min of reaction per OD_600_ of the culture.

### Determination of NAD(P)H/NAD(P)^+^ ratio

Intracellular levels of NAD^+^, NADP^+^, NADH and NADPH were determined by the improved cyclic assay using either ADH (Sigma #A3263) or G6PDH (Sigma #G6378), respectively^53^. The dinucleotides were extracted using cell pellets from 1 mL of culture, cultivated either until the mid-log (OD_600_ of 0.4-0.5) or late-log (OD_600_ of 1.0-1.2) phases. Reduced and oxidised nicotinamide adenine dinucleotides were differentially extracted by treatment with alkali or acid, respectively, followed by extract neutralisation. The assays were performed in 200 μL in a water bath for 30 minutes at 37 °C containing the following components: 0.1 M Tricine–NaOH buffer (pH 8.0); 4.2 mM MTT; 40 mM EDTA (disodium salt); 16.6 mM PES; 5 M ethanol as substrate for alcohol dehydrogenase to determine NADH/NAD^+^ or 25 mM glucose 6-phosphate (dipotassium salt) as substrate for G6PDH to determine NADPH/NADP^+^. To determine NADPH/NADP^+^ and NADH/NAD^+^, 10 µL of a baker’s yeast G6PDH solution (14 units/mL) or 10 µL of a baker’s yeast alcohol dehydrogenase solution (100 units/mL) were added per reaction, respectively. Reactions were stopped by adding 100 μL of 5 M NaCl followed by 5 minutes of ice incubation. The precipitated formazan was centrifuged for 5 min at 14,000 x g and solubilised in 500 μL of 96% ethanol. The formazan was quantified as a function of the absorbance at 550 nm of 200 µL of sample in 96-well plates in a Biotek ELX-800 microplate reader. The standard calibration curve was run in triplicate using up to 30 pmol/assay of either NAD(P)H or NAD(P)^+^ standards.

### Analysis of intracellular ROS levels using flow cytometry

Cells from 1 mL of culture were collected by centrifugation at 14,000 × g for 1 min and then resuspended in 500 μL of PBS buffer supplemented with 1 mM EDTA, 0.01% Tween 20 and 0.1% Triton X-100. Cells were subsequently incubated with 50 μM 2′-7′-dichlorofluorescein diacetate (H_2_-DCFDA) for 30 min at 30 °C in the dark. Control experiments without H2-DCFDA addition were also set up under the same conditions. Treatment with H_2_O_2_ was performed by pre-incubation of cells with increasing concentration of H_2_O_2_ for 30 min at 120 rpm and 30 °C, before addition of H_2_-DCFDA. The samples were analysed by flow cytometry using a BD AccuriTM C5 flow cytometer equipped with a 488 nm argon laser and a 533/30 nm bandpass filter (FL1-H). The median fluorescence intensity was used to determine the intracellular ROS levels.

### Data availability

The datasets generated during and/or analysed during the current study are available from the corresponding author on reasonable request.

## Electronic supplementary material

Supplementary Information
