# Author Correction: The transcriptional regulator NtrC controls glucose-6-phosphate dehydrogenase expression and polyhydroxybutyrate synthesis through NADPH availability in *Herbaspirillum seropedicae*

**DOI:** 10.1038/s41598-020-67125-z

**Published:** 2020-06-22

**Authors:** Euclides Nenga Manuel Sacomboio, Edson Yu Sin Kim, Henrique Leonardo Ruchaud Correa, Paloma Bonato, Fabio de Oliveira Pedrosa, Emanuel Maltempi de Souza, Leda Satie Chubatsu, Marcelo Müller-Santos

**Affiliations:** Department of Biochemistry and Molecular Biology, Laboratory of Nitrogen Fixation, Federal University of Paraná (UFPR), Curitiba, Brazil

Correction to: *Scientific Reports* 10.1038/s41598-017-12649-0, published online 19 October 2017

In the Supplementary Information file originally published with this Article, there were errors in the data and experimental conditions described in Table S3 and Table S4. These errors have been corrected for Tables S3 and S4 in the Supplementary Information that now accompanies the Article.

