# Supplementary material for: The transcriptional regulator NtrC controls glucose-6-phosphate dehydrogenase expression and polyhydroxybutyrate synthesis through NADPH availability in Herbaspirillum seropedicae
Source: Sci Rep. 2017 Oct 19;7:13546. doi: 10.1038/s41598-017-12649-0 (PMC5648810; doi:10.1038/s41598-017-12649-0)
Supplement: Supplementary file 1 — Supplementary Information [file 41598_2017_12649_MOESM1_ESM.docx]

**Supplementary Information**

**The transcriptional regulator NtrC controls glucose-6-phosphate dehydrogenase expression and polyhydroxybutyrate synthesis through NADPH availability in *Herbaspirillum seropedicae***

Euclides Nenga Manuel Sacomboio^1#^, Edson Yu Sin Kim^1#^, Henrique Leonardo Ruchaud Correa^1^, Paloma Bonato^1^, Fabio de Oliveira Pedrosa^1^, Emanuel Maltempi de Souza^1^, Leda Satie Chubatsu^1^, Marcelo Müller-Santos^1*^

**The author Paloma Bonato (PB) designed, performed the transcriptome experiments and analysed the transcriptome data.**

1 – Department of Biochemistry and Molecular Biology, Laboratory of Nitrogen Fixation, Federal University of Paraná (UFPR), Curitiba, Brazil.

^#^Those authors contributed equally

^*^**Corresponding author**: Marcelo Müller-Santos

e-mail: marcelomuller@ufpr.br

tel.: +55-41-3361-1575

fax: +55-41-3266-2042

**Figure Legends**

**Figure S1. Consumption of sugars by *H. seropedicae* SmR1 and *ntrC* mutant in D-glucose, D-fructose and D-xylose as sole carbon sources.** Strains grew in NFbHP medium amended with 25 mM D-glucose (A and D), 25 mM D-fructose (B and E) or 30 mM D-xylose (C and F). Low C/N ratio media had 20 mM NH_4_Cl (blue circles), while high C/N ratio media had 5 mM NH_4_Cl (red circles). Reducing sugars were determined by the dinitrosalicylic acid reduction method[^1^](#_ENREF_1) in three independent samples. Strains were cultivated at 30°C at 120 rpm (orbital shaking).

**Figure S2. The *ntrC* mutant presents increased G6PDH activity.** The G6PDH (A), GDH (B) and ME (C) specific activities were measured in crude extracts of the SmR1 (black bars) and *ntrC* (red bars) strains cultivated at 30°C in NFbHP with 25 mM of glucose and 20 mM of ammonium chloride. The concentration of proteins was measured using the Bradford method[^2^](#_ENREF_2). Where appropriate, statistical significance is shown (* - p-value ≤ 0.05, independent two-sample t-test).

**Figure S1**


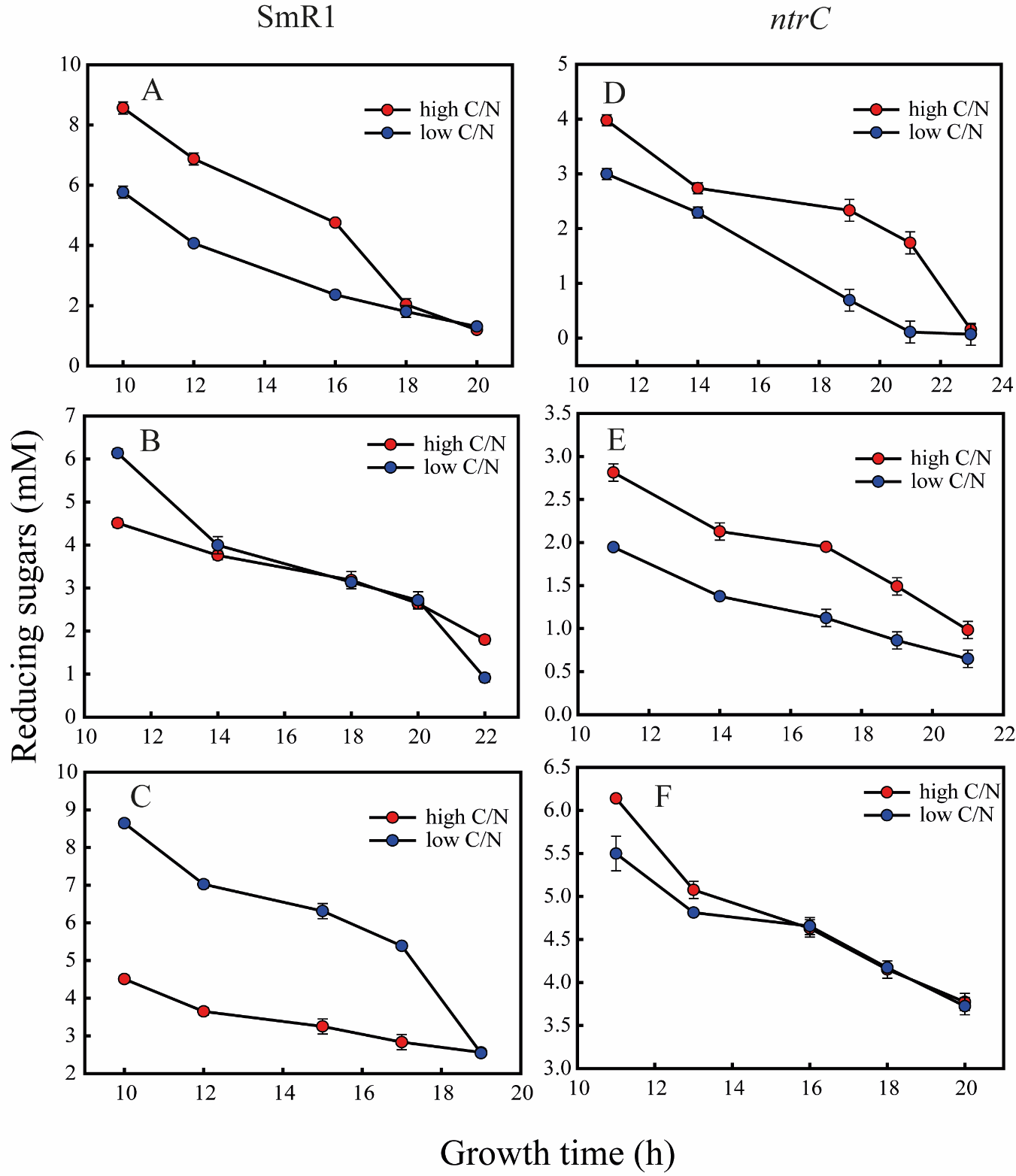


**Figure S2**


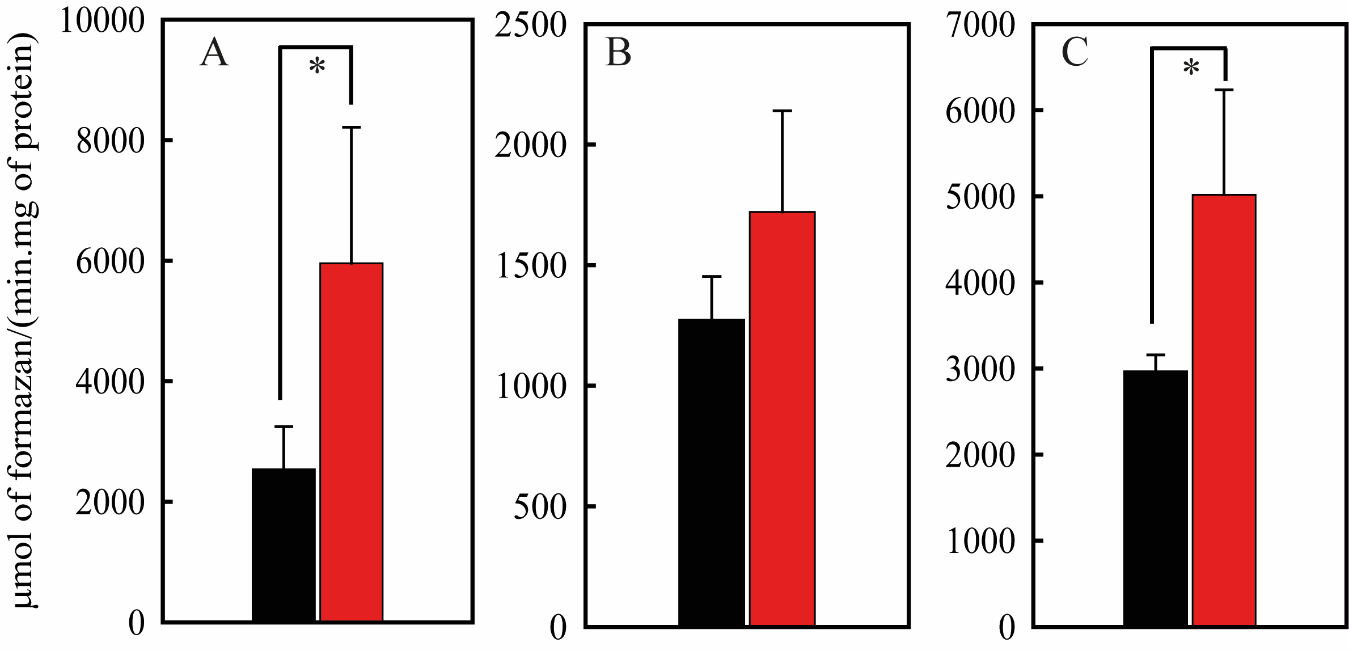


**Table S1. Total biomass and PHB concentration at the maximal PHB production during the growth of *H. seropedicae* SmR1 and *ntrC* mutant.**

| Strain/Growth condition | Cell dry weight (g/L) | PHB (g/L) | % PHB/cdw |
| --- | --- | --- | --- |
| SmR1/glucose and high nitrogen | 0.64 ± 0.01 | 0.19 ± 0.01 | 29.7 |
| ntrC/glucose and high nitrogen | 0.72 ± 0.02 | 0.32 ± 0.02 | 44.5 |
| SmR1/glucose and low nitrogen | 0.49 ± 0.05 | 0.20 ± 0.05 | 40.8 |
| ntrC/glucose and low nitrogen | 0.55 ± 0.05 | 0.29 ± 0.01 | 52.7 |
| SmR1/fructose and high nitrogen | 0.54 ± 0.02 | 0.14 ± 0.02 | 25.9 |
| ntrC/fructose and high nitrogen | 1.09 ± 0.09 | 0.57 ± 0.08 | 52.3 |
| SmR1/fructose and low nitrogen | 0.61 ± 0.03 | 0.19 ± 0.03 | 31.2 |
| ntrC/fructose and low nitrogen | 0.75 ± 0.07 | 0.50 ± 0.08 | 66.7 |
| SmR1/xylose and high nitrogen | 0.70 ± 0.05 | 0.28 ± 0.04 | 40 |
| ntrC/xylose and high nitrogen | 0.71 ± 0.06 | 0.21 ± 0.02 | 29.6 |
| SmR1/xylose and low nitrogen | 0.55 ± 0.02 | 0.18 ± 0 | 32.7 |
| ntrC/xylose and low nitrogen | 0.61 ± 0.01 | 0.36 ± 0.01 | 59 |

**Table S2. The yields of PHB per substrate consumed at the maximal PHB production during the growth of *H. seropedicae* SmR1 and *ntrC* mutant.**

| Strain/Growth condition | PHB (g/L) | substrate consumed  (g/L) | % g PHB/g substrate |
| --- | --- | --- | --- |
| SmR1/glucose and high nitrogen | 0.19 | 4.3 | 4.4 |
| ntrC/glucose and high nitrogen | 0.32 | 4.2 | 7.6 |
| SmR1/glucose and low nitrogen | 0.20 | 4.3 | 4.6 |
| ntrC/glucose and low nitrogen | 0.29 | 4.5 | 7.2 |
| SmR1/fructose and high nitrogen | 0.14 | 4 | 3.5 |
| ntrC/fructose and high nitrogen | 0.57 | 4.3 | 13.2 |
| SmR1/fructose and low nitrogen | 0.19 | 4 | 4.8 |
| ntrC/fructose and low nitrogen | 0.50 | 4.3 | 11.5 |
| SmR1/xylose and high nitrogen | 0.28 | 4.9 | 5.8 |
| ntrC/xylose and high nitrogen | 0.21 | 4.7 | 4.5 |
| SmR1/xylose and low nitrogen | 0.18 | 4.5 | 4 |
| ntrC/xylose and low nitrogen | 0.36 | 4.6 | 7.8 |

**Table S3. Differential transcription of the genes involved in carbohydrate metabolism^a^**

| Gene | Annotation | Locus tag | Fold change (log2)  SmR1 x *ntrC* | p-value  FDR corrected |
| --- | --- | --- | --- | --- |
| *pgi* | glucose-6-phosphate isomerase | Hsero_1099 | -0.26 | 0.92 |
| *zwf* | glucose-6-phosphate dehydrogenase | Hsero_1098 | -0.99 | 0.25 |
| *hexR* | HexR transcriptional regulator family | Hsero_1097 | 0.69 | 0.57 |
| *talB* | transaldolase | Hsero_1096 | 0.01 | 1.00 |
| *edd* | phosphogluconate dehydratase | Hsero_1100 | -0.19 | 0.95 |
| *eda* | keto-deoxy-phosphogluconate aldolase | Hsero_1101 | -0.63 | 0.78 |
| *pgl* | 6-phosphogluconolactonase | Hsero_3149 | -0.56 | NA^b^ |
| *tktA1* | transketolase | Hsero_0303 | 0.30 | 0.88 |
| *tktA2* | transketolase | Hsero_0304 | 0.51 | 0.72 |
| *gpmA* | phosphoglyceromutase | Hsero_0163 | 0.20 | 0.93 |
| *eno* | enolase | Hsero_2770 | 0.52 | 0.62 |
| *pykA* | pyruvate kinase | Hsero_0671 | 0.25 | 0.89 |
| *gltA* | citrate synthase | Hsero_2970 | -0.54 | 0.41 |

^a^ The bacterial strains were grown in NFb-malate in 10 mM NH_4_Cl to OD_600_ 0.4. The cultures were collected by centrifugation (12.000 x g, 2 min, room temperature) and the total RNA was extracted using Trizol®. Ribosomal RNA (rRNA) was removed using the Ribo-Zero™ rRNA Removal kit (Gram-negative bacteria) (Illumina - Epicentre, Madison, WI, USA). cDNA libraries were constructed using Ion Total RNA-Seq Kit v2 (Applied Biosystems, Waltham, MA, USA) and sequenced in an Ion Proton™ Sequencer (Thermo Fisher Scientific, Waltham, MA, USA). The quality check of the data and the reads mapping were performed in the CLC Genomics Workbench 7.0. The fold change was calculated by DESeq 2.0 dividing the mutant value (*ntrC*) by the parental value (SmR1). The fold changes are the average of two independent experiments. ^b^ Not available.

**Table S4. Differential transcription of the genes involved in PHB metabolism^a^**

| Gene | Annotation | Locus tag | Fold change (log2)  SmR1 x *ntrC* | p-value  FDR corrected |
| --- | --- | --- | --- | --- |
| *phaA1* | β-ketothiolase | Hsero_0239 | -0.28 | 0.88 |
| *phaB1* | acetoacetyl-CoA reductase | Hsero_2998 | -0.34 | 0.87 |
| *phaC1* | PHA synthase | Hsero_2999 | -0.08 | 0.96 |
| *phaP1* | phasin | Hsero_1639 | -2.27 | 8.6 x 10^-10^ |
| *phaP2* | phasin | Hsero_4759 | -0.95 | 0.45 |
| *phaR* | transcriptional repressor of phasin transcription | Hsero_2997 | -0.08 | 0.01 |

^a^ The bacterial strains were grown in NFb-malate in 10 mM NH_4_Cl to OD_600_ 0.4. The cultures were collected by centrifugation (12.000 x g, 2 min, room temperature) and the total RNA was extracted using Trizol®. Ribosomal RNA (rRNA) was removed using the Ribo-Zero™ rRNA Removal kit (Gram-negative bacteria) (Illumina - Epicentre, Madison, WI, USA). cDNA libraries were constructed using Ion Total RNA-Seq Kit v2 (Applied Biosystems, Waltham, MA, USA) and sequenced in an Ion Proton™ Sequencer (Thermo Fisher Scientific, Waltham, MA, USA). The quality check of the data and the reads mapping were performed in the CLC Genomics Workbench 7.0. The fold change was calculated by DESeq 2.0 dividing the mutant value (*ntrC*) by the parental value (SmR1). The fold changes are the average of two independent experiments.

**References**

1 Miller, G. L. Use of Dinitrosalicylic Acid Reagent for Determination of Reducing Sugar. *Analytical chemistry* **31**, 426-428, doi:10.1021/ac60147a030 (1959).

2 Bradford, M. M. A rapid and sensitive method for the quantitation of microgram quantities of protein utilizing the principle of protein-dye binding. *Analytical Biochemistry* **72**, 248-254, doi:<http://dx.doi.org/10.1016/0003-2697(76)90527-3> (1976).
